# International Pocket Medical Formulary

**Published:** 1889-04

**Authors:** 


**International Pocket Medical Formulary.** With an appendix containing Posological Table Formulæ for Inhalations, Suppositories, Nasal Douches, Eyewashes, and Gargles; Hypodermic Formulæ; Table of Hypodermic Medication; Use of Thermometer in Disease; Poisons and their Antidotes; Postmortem and Medico-legal Examinations; Artificial Respiration; Ligation of Arteries; Obstetrical Table; Urinalysis; Differential Diagnosis of Eruptive, Typhoid, and Typhus Fevers; Tables of Pulse Temperature, Respiration; Motor Points, etc. By C. SUMNER WITHERSTINE, M. S., M. D., Associate Editor "Annual of the Universal Medical Sciences;" late House Surgeon Charity Hospital, New York; Visiting Physician Home for the Aged (Little Sisters of the Poor), Germantown, Philadelphia. Pp. 269; price, $2.00 net. Philadelphia and London: F. A. Davis, publisher. 1888.

This is a neatly gotten-up little volume of 1658 formulæ, taken, for the most part, from well-known therapeutic works and from the pages of the "Annual of the Universal Sciences. It is indexed so that each subject can be referred to in alphabetical order, and is interleaved for such additions as the reader may desire to make. The appendix contains a variety of useful information upon the important points alluded to in the title. The author states in the preface that he offers it to the profession in the hope that it may meet a demand made known to him by many of its members. We cannot approve of the present methods, adopted by many physicians, of resorting to ready-made formulæ in the application of remedies to the treatment of disease; and yet, it is sometimes important that such prescriptions as have been well established in their usefulness be preserved for reference. This little volume serves such a purpose better than any one we have seen, and in that sense we commend it to such as may seek to utilize the information which it contains. It is neatly bound in flexible morocco, and is offered at a reasonable price.

